# Isokinetic Strength Test of Muscle Strength and Motor Function in Total Knee Arthroplasty

**DOI:** 10.1111/os.12699

**Published:** 2020-05-21

**Authors:** Xiao‐fei Wang, Zhen‐hua Ma, Xue‐ren Teng

**Affiliations:** ^1^ Dalian Medical University Dalian China; ^2^ Department of Orthopaedic Surgery Qingdao Municipal Hospital Qingdao China

**Keywords:** Function evaluation, Knee, Osteoarthritis, Range of motion

## Abstract

**Objective:**

To use isokinetic strength testing system to test and analyze the relationship between changes in muscle strength before and after knee replacement in patients undergoing total knee arthroplasty (TKA).

**Methods:**

A total of 200 patients with advanced knee osteoarthritis treated from June 2018 to June 2019 were selected for TKA. The patient's isokinetic muscle strength test was performed in the first, third, and the sixth month before and after the operation. The knee hamstring peak torque (PT value), quadriceps peak torque (PT value), and total work were mainly measured. The knee joint was evaluated at the hospital for special surgery score, range of motion and other knee function standards, and then healthy limbs and normal people were tested with the same method. Statistical data was used to analyze and deal with the data, evaluate the muscle strength and motor function changes with time progressing, then compare the differences to the healthy limb. From *P* < 0.05, we can see that the differences have some statistical significance. The influences that TKA has on motor function changes of lower limbs were also observed.

**Results:**

Among the 200 subjects, 162 completed all follow‐up tests, and the remaining 38 were lost to follow‐up for various reasons. The rate of loss of follow‐up was approximately 19%. The isokinetic muscle strength test system and the knee joint function scoring standard were used to record the knee joint muscle strength and function changes before and after knee joint replacement. Statistical analysis was performed to show the knee joint hamstring muscle force and quadriceps muscle strength and joint mobility in the first month after the surgery. The knee joint muscle strength and joint mobility were significantly improved after the third month after the surgery, but there were still some differences compared with normal people. The knee function index was significantly improved in the sixth month after operation (*P* < 0.05), and there were no significant differences compared with normal people.

**Conclusions:**

Knee joint strength and knee function after TKA are significantly improved compared with preoperative function, which is of great significance for the treatment of knee osteoarthritis. The constant velocity muscle strength test system has the advantages of safety, accuracy, repeatability and easy operation. It is a good method to evaluate the knee joint's muscle strength and function after the knee joint replacement.

## Introduction

Knee osteoarthritis (KOA) is a disease commonly seen in middle‐aged and elderly people based on degenerative pathological changes of the knee joint and characterized by degeneration of articular cartilage and secondary bone hyperplasia. There is pain and swelling of the knee joint, changes in muscle strength of the lower limbs, and knee stiffness. Loss of joint mobility may occur in the late stage, which significantly reduces the quality of life of patients, and its incidence continues to increase^1^. Due to the development of the disease to the late stage, there are many different degrees of deformity, making it the main disabling disease of the skeletal and muscular system of the elderly. Common pathogenic factors include age, abnormal biomechanics, body mass index, and genetics, among which age is the main risk factor. Auxiliary examinations are often based on the full length X‐ray examination of both lower limbs. X‐rays often show narrowing of the joint space, osteophyte formation at the joint edges, subchondral bone sclerosis, and cystic changes.

The focus of treating knee osteoarthritis is to prevent the cartilage from aggravating wear and tear, which is divided into conservative treatment and surgical treatment^2^. Conservative treatment is mainly applicable to patients with mild symptoms, and can be administered as oral medication, local injection or physical therapy. Total knee arthroplasty (TKA) is the main surgical method for surgery. It is suitable for advanced patients who have failed regular conservative treatment. It can significantly improve knee mobility, relieve joint pain, restore lower limb force lines, and improve knee function^3^. The knee joint is one of the main weight‐bearing joints, so the probability of degenerative changes is high. If the pain associated with osteoarthritis cannot be controlled by conservative treatment, total knee replacement may be the most appropriate treatment option. A variety of surgical techniques and prosthetic components are currently available, giving plastic surgeons the opportunity to replace part or all of the surface of the knee joint depending on the extent of the disease. With medical development, TKA is now more mature than traditional techniques and has benefits such as requiring less muscle anatomy, shorter incisions, less blood loss, and shorter hospital stays. The accuracy of the surgical technique and effective rehabilitation after the operation are also successful.

The development of modern TKA has always been aimed at improving knee function and prolonging the life of the implant. With the improvement of modern surgical techniques, the expectations and demands of patients are also increasing. At present, medical practitioners are constantly improving surgical techniques to improve the recovery of knee function and clinical treatment results. However, while we pay attention to the development of surgical technology, we often ignore the strength of the patients' knee joints. The strength of the muscles around the knee has an inseparable relationship with the patients' daily activities. Patients often suffer from stiff knee joints, atrophy of surrounding muscles, and a decline in muscle strength of the lower limbs, which is mainly manifested by simultaneous decline in muscle strength of the extensor knee (quadriceps) and flexion of the knee (hamstrings)^4^. However, the decline of knee extension and flexion muscle strength is an important clinical manifestation of sarcopenia and knee joint dysfunction, which can lead to a decline in knee mobility and increase the degree of osteoarthritis. The speed and extent of the damage continues to increase. Osteoarthritis of the knee joint can cause weakness of the knee joint, which is mainly in the extension of the knee, and weakness of the knee extensor muscles will cause the gait speed of the lower limbs to significantly slow down, so the knee extension force is an important indicator of gait^5^. And with the aging of the population, the loss of skeletal muscle mass and strength is a sign of more and more sarcopenia and dyskinesia. The quadriceps and hamstrings of the knee joint are important components, and can even cause difficulty in walking the lower limbs. These risks associated with skeletal muscle structure and functional aging also lead to increased morbidity and mortality of knee osteoarthritis^6^. Therefore, in order to improve knee function, TKA is particularly important for restoring muscle strength around the knee joint^7^.

However, TKA is only a prerequisite to restore the normal or basic normal function of the joint, and postoperative rehabilitation is an important and key part. Active rehabilitation training after replacement can effectively shorten the length of hospital stay, alleviate the symptoms of knee discomfort, and promote knee function recovery^8^. Therefore, how to accurately assess the functional rehabilitation process of patients after surgery has always been an important issue in clinical research. The TKA will cause corresponding changes in surrounding joint forces and muscle strength. Therefore, using accurate quantitative measurements of changes in muscle strength around the knee joint can help identify osteoarthritis of the knee joint and the changes in kinematics and kinematics of the knee joint after surgery; at the same time, it can evaluate and correct knee biomechanics for the early postoperative period. This abnormality has very significant clinical significance^9^. In addition, TKA is a treatment method aimed at restoring the function of the knee joint and improving the patient's quality of life. Although there are relevant reports of changes in muscle strength after total knee replacement, there is currently no follow‐up of changes in muscle strength and function around the knee after TKA. We can determine the degree of recovery of knee extension, knee flexion, and knee function in patients at various stages after knee replacement by isokinetic strength measurement of the knee joint, so as to formulate their rehabilitation plan according to the specific conditions of each stage, and promote recovery of muscle strength, mobility and function around knee joints after TKA.

Through this project, we can further understand the relationship between TKA and changes in muscle strength around the knee joint, and clarify the recovery time necessary for restoring the balance of bilateral knee muscle strength. We objectively analyze the role of replacement surgery in promoting knee joint recovery in patients. In this study, the isokinetic strength test system was used to monitor the changes in knee muscle strength before and after surgery for patients undergoing TKA. Statistical analysis was used to study and analyze the effect of TKA on the patient's knee. The relationship between the recovery time of muscle strength around the joints and the change in motor function of the lower limbs was analyzed. Through the study of this subject, we can also compare the changes of the muscle strength around the affected knee and the muscle strength around the healthy knee before and after surgery. We can also observe the effect of TKA on the changes of lower limb motor function. By instructing patients in the rehabilitation treatment after TKA, more rehabilitation methods for restoring the strength of the quadriceps and hamstring muscles are obtained. Through multiple comparison methods, a more practical evaluation method can be obtained.

## Materials and Methods

This study was performed at Qingdao Municipal Hospital from June 2018 to June 2019.

Research subjects selected for this study were 200 patients with advanced knee osteoarthritis and TKA who were treated from June 2018 to June 2019. All patients in this experiment were informed of the detailed topic and understanding of the study and all signed the informed consent for this study. The trial has been approved and supervised by the relevant unit's ethics review committee.

#### *Inclusion Criteria*

Inclusion criteria are as follows: (i) middle‐aged and elderly patients (age >50 years) who meet the KOA diagnostic criteria; (ii) symptoms do not ease after regular conservative treatment; (iii) there is no history of trauma and surgery on the affected knee joint; (iv) there is no obvious degenerative disease on the contralateral knee joint; and (v) patients undergoing TKA surgery.

#### *Exclusion Criteria*

Exclusion criteria are as follows: (i) patients with contralateral limbs or who have undergone surgery; (ii) patients with muscle disorders such as myasthenia gravis, progressive malnutrition, and periodic paralysis; (iii) patients with deep vein thrombosis of the lower limbs during perioperative period; (iv) patients with cerebrovascular disease or with severe medical diseases; and (v) people who need knee replacement surgery due to other knee joint diseases.

### *Device Introduction*

The introduction of knee implants includes artificial knee joint prostheses including femoral side prosthesis, tibial side prosthesis, bone cement, disposable flusher, and so on. The types of prostheses used are divided into PFC, PS150, ATUUNE, and other types, and the above devices are provided by Johnson & Johnson.

### *Surgical Methods*

All study subjects were operated on by the same group of surgeons. All patients underwent TKA under epidural and subarachnoid block anesthesia. After successful anesthesia, the patient was supine on the operating table, and a tourniquet of an airbag was attached to the proximal thigh. Polyvinyl Pyrrolidone iodine disinfects the skin of the operating area, a sterile towel is spread, and the affected limb is raised. The balloon tourniquet is inflated with a balloon pressure of 400 mm Hg.

A longitudinal incision was made in the front and middle of the affected knee, and the epidermis, subcutaneous tissue, and superficial fascia were successively drawn about 15 cm in length. At the superficial fascia, the flap was peeled medially to the medial edge of the patella. The quadriceps and the patellar tendon tissue were expanded, and then the joint capsule, synovial tissue, and submental fat pad were separated, and the patella was turned outward. It could be detected during the operation that the articular cartilage of the patella and the medial condyle of the femur were worn, the subchondral bone was exposed, and the joint synovium was significantly thickened and congested.

The anterior and posterior cruciate ligaments were removed, and the proximal tibia was pulled out with the externally rotated calf. Part of the meniscus was excised along the edge of the joint capsule and separated along the tibia to the posteromedial angle of the lateral platform.

The anterior angle of the lateral meniscus was cut, the entire lateral meniscus was excised along the edge of the articular capsule, the tibial joint of the articular capsule was cut off, and it was peeled to the posterolateral angle of the lateral platform along the tibial periosteum.

The osteotome was used to remove excess bone and drilled through the bone marrow cavity 1 cm at the cruciate ligament stop. The positioning rod was inserted in the femoral bone marrow cavity, performing a 5° osteotomy of the distal femur, and the thickness of the osteotomy was 9 mm. The femoral condyle was installed, and the femoral condyle pre‐reference method was used to measure the femoral prosthesis size. With external rotation of 3°, the femoral osteotomy template was installed in order to perform osteotomy, and the central iliac box osteotomy mold was installed again to perform the central iliac box osteotomy.

After the tibial positioning rod is installed, the osteotomy is carried out at an angle of 0°, and the thickness of the osteotomy is 11 mm. The femoral prosthesis test model was installed, the knee joint and flexed and stretched, and it was found that the joint straightness was 0° and the flexion reached 120°. There was no slack in the internal and external movements of the knee joint in the extended and 90° flexed knee positions. The excess bone around the patella was removed and the patella was cleaned. Around the synovial tissue, the cartilage surface is cut off from the inside out.

The joint was reset, and the knee joint was flexed and stretched to see that the patella prosthesis had a good running trajectory. The rotation center of the osteotomy prosthesis was located at 1/3 of the medial tibial tubercle and marked with an electric knife. Take out the prosthesis test model, drill the central column hole on the proximal osteotomy surface of the tibia, repeatedly clean the joint cavity, and wipe dry with gauze cotton pad.

Gentamicin bone cement was evenly applied to the tibial plateau and femoral condyle, and the joint prosthesis was hammered. The patella was treated at the same time, a gasket was placed and squeezed for 12 min. After the bone cement hardened, the gasket was removed to clean up the excess bone cement, and a good local anesthetic was injected around the joint capsule.

A shim was placed according to the size of the tibial prosthesis, the knee joint was extended and flexed to see that the knee joint was in good motion, sutures were stitched layer by layer, and elastic bandage compression bandage was applied.

### *Postoperative Management*

The patient's blood pressure, heart rate, blood oxygen saturation, and respiratory vital signs were closely monitored with electrocardiogram (ECG) monitoring equipment after operation, and the urinary tube was removed to exercise the urination function on the second day after operation. Blood was drawn for three consecutive days after operation to observe changes in blood routine, electrolytes and other indicators, and symptomatic treatment was performed according to the specific situation of the patient.

Antibiotics were used prophylactically postoperatively until the third day postoperatively. Conventional intravenous and oral analgesics were used in combination to prevent pain after knee incision and knee rehabilitation. Low‐molecular‐weight heparin was applied to prevent deep vein thrombosis of the lower extremity 6 h after operation, and was changed to oral anticoagulants after discharge to 1 month after operation. On the third day after the operation, the knee X‐rays were reviewed to see if the prosthesis is in good position.

Postoperatively, the affected limb was raised to prevent edema. After the anesthesia was restored, straightening exercises, ankle pump training, and quadriceps relaxation training were performed, and straight leg lifting exercises were performed on the first day after surgery. Rehabilitation doctors will guide the patients to perform rehabilitation exercises after surgery. After removing the elastic bandage on the second day after surgery, they can assist the walker to walk down and perform knee extension and flexion exercises. Apply ice for 15–20 min after each extension and flexion exercise. If the knee joint is swollen or painful, apply ice again every 2 h.

After discharge, the patient recovered on his own and strengthened knee flexion and extension. The flexion and extension angle should be greater than 120° 4 weeks after surgery. Encourage patients to gradually carry weight and increase independent walking distance to restore muscle strength and gait around the knee joint.

### *Isokinetic Strength Test Method*

The isokinetic muscle strength test system (ISOMED2000) was used to perform isokinetic muscle strength tests on the affected and healthy limbs at various time points before and after knee replacement to obtain specific data analysis research results.

Adjust the back of the instrument: According to the instructions of the isokinetic strength test system, first adjust the back of the instrument, including the angle adjustment of the training chair back, the adjustment of the seat back, the angle adjustment of the seat cushion, and the pull‐out of the leg support. So that the patient is placed in a comfortable position.

The patient's posture is fixed: the knee joints on both sides are placed in front of the instrument and both hands can be placed on the armrests on both sides so that the handle can be held during the test to maintain its stability. Rotation adjustment dynamometer includes coarse adjustment of rotation angle, fine adjustment of rotation angle, tilt angle adjustment, height adjustment, and rotation arm position adjustment. The angle of the rotating arm is preferably 30°–40°, and the action should be light during operation. There should be no patients or other personnel in the range of the dynamometer's rotating arm to avoid injury.

Laser positioning: The subject's measured limb is placed on the dynamometer, and the patient is instructed to perform knee extension and flexion exercises, and the laser is positioned at the midpoint of the knee joint movement, and the measured limb is placed so as not to hinder knee flexion and extension. Activities are appropriate.

### *Clinical Assessment*

The ISOMED2000 instrument was used to test the isokinetic muscle strength of the knee joints of both sides of the study subject, and the balance was calibrated and adapted to exercise before the test. Patients were tested for isokinetic strength of the affected knee joint before surgery, 1 month, 3 months, and 6 months after surgery, and the uninjured knee joint was tested in the same way. The patient is asked to rest for 15 min after each test, and record the average after repeating the test three times. The recorded angular velocity was 60°/s. Test and record the peak hamstring strength (PT value), quadriceps peak torque value (PT value), total knee flexion effort (TW), knee extension effort (TW) and other indicators. The knee joint HSS score was used to evaluate knee function according to the state of the affected knee before and after the operation.

#### *Peak Torque (PT)*

The PT value refers to the maximum acting torque released by the muscle during contraction. It is the torque value at the highest point of the torque curve and represents the maximum muscle force produced by muscle contraction. The unit is N·m. The test was repeated three times to record the average. Using the isokinetic test system, the peak force distance of the knee hamstring muscles (knee flexion) and the peak torque value of the quadriceps muscles (knee extension) are reliable test indicators for measuring the muscle strength around the knee joint. After system analysis, the measurement results will be displayed on the display. The statistical software was used to compare whether the PT of knee extension and knee flexion before and after surgery was statistically significant.

#### *Total Work (TW)*

The TW value refers to the total work done by the knee joint once each flexion and extension is performed. According to the definition of physics, work is the product of force and the distance traveled by an object. So it is not only related to the muscle strength of the lower limb muscle group, but also to the distance of the force. The TW value reflects the actual motion of the knee joint, and its unit is joule (J). After isokinetic testing, the TW values of flexors and extensors of the patients before and after TKA were recorded. Through statistical comparison, we calculated whether the TW of the patients before and after surgery was statistically significant.

#### *Hospital for Special Surgery (HSS)*

This HSS score is an assessment of changes in knee function^10^. The HSS score is a scoring system proposed by the Hospital Special Surgery (HSS) in 1976. It is very accurate for evaluating the recovery of joint function before and after TKA and for comparison before and after evaluation. Especially, the early functional evaluation of surgery has obvious advantages. This can be a good assessment of the knee function. The score is out of 100 points: excellent is greater than 85 points; good is 70–84 points; intermediate is 60–69 points; ad poor is less than 59 points.

### *Statistical Analysis*

SPSS25.0 statistical software system was used to perform statistical analysis on the collected results. The measurement data obeyed the normal distribution. The data was expressed in the form of mean ± standard deviation (mean ± SD). The statistical method used repeated measures analysis of variance and multivariate analysis of variance. With *α* = 0.05 as the test level, the difference was statistically significant at *P* < 0.05.

## Results

Of the 200 knee osteoarthritis subjects included in this subject, 162 completed all follow‐up tests, and the remaining 38 patients were lost to follow‐up or failed to complete all follow‐up tests for various reasons. The rate of lost follow‐up was about 19%.

### *Baseline Information*

Of the 200 patients included in the study, 162 patients completed all follow‐ups, including 69 male patients (42.6%) and 93 female patients (57.4%). The age of patients admitted was 50–82 years, with an average of 64.64 ± 5.98 years. Among them, 45 were middle‐aged patients aged 50–60 years (28%), 99 were aged 60–70 years (61.1%), 18 were aged 70 years or older (11.1%); 89 were left knee replacement, and 73 were right knee replacement.

### *Impact of Knee Extensor Muscle Strength Before and After Knee Replacement*

After univariate repeated measurement analysis of variance, there was a significant difference in the extensor muscle strength of the affected side during multiple surgical periods (*F* = 3949.874, *P* < 0.01). After multiple comparisons, it was found that knee extension muscle strength before operation <1 month after operation <3 month after operation <6 month after operation. Multiple comparisons revealed that the extensor knee muscle strength before and 1 month after surgery was less than that at 3 months after surgery. After two‐way repeated measurement analysis of variance, there was a difference in muscle strength between the affected side and the healthy side (*F* = 4312.204, *P* < 0.01). Using multivariate analysis of variance, it was found that the extensor knee muscle strength of the affected side was less than that of the healthy side before surgery, 1 month after surgery, and 3 months after surgery (*F* = 5297.406, *P* < 0.01; *F* = 5489.536, *P* < 0.01; *F* = 1951.911, *P* < 0.01). At 6 months postoperatively, the muscle strength of the knee extensor on the affected side improved significantly compared to other periods, and was close to the muscle strength on the healthy side (*F* = 14.812, *P* < 0.01).

It can be concluded that no significant changes were seen in the knees of the healthy side at various times. The knee extensor muscle strength of the affected side changed significantly in the two periods of 1 to 3 months and 3 to 6 months after surgery, and it showed a steady upward trend overall. Among them, it is more obvious at 3–6 months, which indicates that the knee extensor muscle strength improves rapidly during this period (Table 1).

**Table 1:** Isokinetic muscle peak knee extension torque (N•m, ^−^x ± s, *n* = 162)

Sides	Before surgery	1 month after operation	3 month after operation	6 month after operation	*F* value	*P* value
Affected side	44.91 ± 9.52	46.16 ± 8.95	66.27 ± 9.54	91.02 ± 6.92	3949.874	0.000
Healthy side	92.39 ± 6.15	92.52 ± 5.81	93.33 ± 5.89	92.63 ± 5.32	4.832	0.005
*F* value	5297.406	5498.536	1951.911	14.812	4312.204	0.000
*P* value	0.000	0.000	0.000	0.000		

### *Impact of Knee Flexion Strength Before and After Knee Replacement*

After univariate repeated measurement analysis of variance, there was a difference in the flexion of the knee flexion force between different surgical methods on the ipsilateral side (*F* = 5270.496, *P* < 0.01). After multiple comparisons, it was found that the knee flexion muscle strength before operation <1 month after operation <3 months after operation <6 months after operation. There was no difference in flexor knee muscle strength between different surgical methods on the healthy side (*F* = 1.371, *P* > 0.05). After two‐way repeated measurement analysis of variance, there was a difference in muscle flexion between the affected and uninvolved knees (*F* = 633.277, *P* < 0.01). Using multivariate analysis of variance, it was found that the muscle strength of the knee flexion on the affected side was less than that on the healthy side before surgery, 1 month after surgery, 3 months after surgery, and 6 months after surgery (*F* = 1268.123, *P* < 0.01; *F* = 1358.704, *P* < 0.01; *F* = 221.622, *P* < 0.01; *F* = 9.49, *P* < 0.01).

We can conclude that the flexion strength of the knee flexion on the affected side changes after 3–6 months. Knee flexor muscle strength showed a significant upward trend as a whole after 1 month. At 6 months after operation, the hamstring muscle strength of the operative side significantly improved compared with that before operation, 1 month after operation, and 3 months after operation, and was close to that of the contralateral knee (Table 2).

**Table 2:** Knee peak isokinetic muscle flexion peak knee moment (N•m, ^−^x ± s, *n* = 162)

Sides	Before surgery	1 month after operation	3 month after operation	6 month after operation	*F* value	*P* value
Affected side	33.34 ± 5.73	34.58 ± 5.41	52.29 ± 5.61	62.14 ± 3.63	5270.496	0.000
Healthy side	63.66 ± 9.43	63.75 ± 8.61	63.78 ± 7.84	64.19 ± 7.36	1.371	0.255
*F* value	1268.123	1358.704	221.622	9.49	633.227	0.000
*P* value	0.000	0.000	0.000	0.002		

### *Changes in Total Knee Extension*

After univariate repeated measurement analysis of variance, there was a difference in the total amount of knee extension with different surgical methods on the affected side (*F* = 2516.354, *P* < 0.01). After multiple comparisons, it was found that total knee extension work (TW) before surgery <1 month after surgery <3 months after surgery <6 months after surgery. After two‐way repeated measurement analysis of variance, the relative ratios of total knee effort between the affected side and the healthy side were significantly different (*F* = 2514.762, *P* < 0.01). Using multivariate analysis of variance, it was found that the total amount of knee extension on the affected side was lower than that on the healthy side before surgery, 1 month after surgery, 3 months after surgery, and 6 months after surgery (*F* = 3051.079, *P* < 0.01; *F* = 2920.533, *P* < 0.01; *F* = 818.563, *P* < 0.01; *F* = 7.516, *P* < 0.01).

We can conclude that the knee extension TW of the affected side changed after 6 months. The difference at 1 month after surgery was smaller than that before surgery, and then showed a significant upward trend. At 6 months after surgery, the knee extension TW was close to the level of the uninjured knee joint (Table 3).

**Table 3:** Total knee extension of knee joint (J, ^−^ *x* ± *s*, *n* = 162)

Sides	Before surgery	1 month after operation	3 month after operation	6 month after operation	*F* value	*P* value
Affected side	381.32 ± 65.46	392.82 ± 63.75	542.49 ± 58.04	636.99 ± 51.81	2516.354	0.000
Healthy side	643.79 ± 52.18	641.15 ± 52.22	633.33 ± 53.17	642.63 ± 50.91	23.292	0.000
*F* value	3051.079	2920.533	818.563	7.516	2514.762	0.000
*P* value	0.000	0.000	0.000	0.007		

### *Changes in Total Knee Flexion*

After univariate repeated measurement analysis of variance, there was a difference in the total amount of flexed knees with different surgical methods on the affected side (*F* = 5068.374, *P* < 0.01). After multiple comparisons, it was found that total knee flexion effort (TW) before surgery <1 month after surgery <3 months after surgery <6 months after surgery. After multiple comparisons, it was found that there was a difference in the total knee flexion work of the healthy side of the knee joint during different surgical time periods> 1 month after surgery and 6 months after surgery (*F* = 5.330, *P* <0.01). Repeated measurement by two‐factor analysis of variance showed that there was a difference in total knee flexion between the affected side and the healthy side (*F* = 3854.765, *P* < 0.01). Using multivariate analysis of variance, it was found that the total amount of knee flexion on the affected side was less than that on the healthy side before surgery, 1 month after surgery, 3 months after surgery, and 6 months after surgery (*F* = 5750.849, *P* < 0.01; *F* = 5100.803, *P* < 0.01; *F* = 760.277, *P* < 0.01; *F* = 25.365, *P* < 0.01;).

It can be seen that the knee flexion TW on the affected side changed after 6 months. The difference at 1 month after surgery was smaller than that before surgery, and then showed a significant upward trend. At 6 months after surgery, the knee flexion TW was close to the level of the knee flexion on the contralateral side, and the change was significant. (Table 4).

**Table 4:** Total knee flexion of the knee joint (J, ^−^ *x* ± *s*, *n* = 162)

Sides	Before surgery	1 month after operation	3 month after operation	6 month after operation	*F* value	*P* value
Affected side	359.3 ± 64.59	368.94 ± 63.91	556.43 ± 56.24	633.34 ± 50.29	5068.374	0.000
Healthy side	643.83 ± 47.86	640.39 ± 49.34	640.87 ± 48.6	640.1 ± 47.85	5.330	0.001
*F* value	5750.849	5100.803	760.277	25.365	3954.765	0.000
*P* value	0.000	0.000	0.000	0.000		

### *Changes of Knee* *HSS* *Score Before and After Replacement*

Using single factor repeated measurement analysis of variance, the HSS scores of the affected side were significantly different in multiple surgical time periods (*F* = 12057.720, *P* < 0.01). After multiple comparisons, it was found that at <1 month after surgery, <3 months after surgery, and <6 months after operation there was no significant difference in HSS between different surgical methods on the healthy side (*F* = 2.253, *P* > 0.05). After two‐factor repeated measurement variance calculation, the HSS of the affected side and the healthy side were significantly different (*F* = 17655.532, *P* < 0.01). Using multivariate analysis of variance, it was found that the HSS of the affected side was smaller than that of the healthy side before surgery, 1 month after surgery, 3 months after surgery, and 6 months after surgery (*F* = 24239.661, *P* < 0.01; *F* = 24879.001, *P* < 0.01; *F* = 4247.575, *P* < 0.01; *F* = 10.872, *P* < 0.01;).

It can be known that the HSS score of the affected knee joint is statistically significant compared with that before surgery. Especially in the period of 3–6 months, the change is more significant, and the overall trend shows a steady upward trend. Over time, the difference between the HSS score of the knee joint and the healthy side narrowed within 6 months after the operation. It was close to the healthy side at 6 months after operation, indicating that the knee joint mobility and other indicators were significantly better than before (Table 5).

**Table 5:** HSS score of knee joint function(*x* ± *s*, *n* = 162)

Sides	Before surgery	1 month after operation	3 month after operation	6 month after operation	*F* value	*P* value
Affected side	35.33 ± 3.88	38.24 ± 3.76	65.48 ± 4.65	87.41 ± 2.67	12057.720	0.000
Healthy side	89.04 ± 2.47	89.76 ± 2.01	89.91 ± 1.9	89.21 ± 6.62	2.253	0.128
*F* value	24239.661	24879.001	4247.575	10.872	17655.532	0.000
*P* value	0.000	0.000	0.000	0.001		

## Discuss

According to the current aging changes in the social population, the number of knee osteoarthritis patients has also increased significantly. Daily activities of patients with mild lesions can cause knee pain, and severe cases significantly reduce the quality of life of patients due to decline in knee function. If the patient is not treated in time and prevents the disease from progressing further, the muscle strength and motor function around the knee joint will be severely reduced, and eventually the knee joint will develop into advanced disease. Now medical technology has significantly improved and TKA has become a mature surgical method. Many patients have achieved satisfactory clinical results after surgical treatment and are accepted by more and more patients. However, patients with advanced knee osteoarthritis often have stiff knee joints, atrophy of surrounding muscles, and subsequent decline in muscle strength. Whether TKA can restore muscle strength and functional changes around the knee joint has always been our focus.

### *KOA* *Causes Decreased Muscle Strength Around the Knee Joint*

As we all know, KOA is a common degenerative disease of the knee joint. There are many risk factors that can cause KOA, which are divided into unchangeable and changeable factors. Among them, genetics, gender and age are unchangeable risk factors; body mass index, lack of exercise, and a history of knee trauma are modifiable risk factors. These are factors that may increase the likelihood of osteoarthritis. Gender and age are the strongest predictors of osteoarthritis, and the risk of knee osteoarthritis is significantly increased in obese people and patients with a history of knee trauma. With the development of research, more and more scholars have realized that the peripheral muscle strength of KOA patients will change, and the main impacted peripheral muscle strength is quadriceps and hamstring muscles. These two muscles affect the activities of knee extension and knee flexion, of which the knee extension is more obvious.

Changes in the muscle strength of knee extension and flexion directly affect knee function and reduce the quality of life of patients. Relevant research reports that there are many reasons why knee osteoarthritis can reduce muscle strength in patients. An important cause of impaired muscle function is the presence of articular muscle depression (AMI) or the inability to fully activate the quadriceps^11^. Common symptoms of quadriceps AMI and KOA such as swelling, pain, stiffness of the knee joint are related to afferent joint damage and changes in joint receptor discharge. Inflammation of joints and joint relaxation can also lead to the occurrence of joint muscle suppression of the quadriceps femoris^12^.

Luc‐Harkey *et al*.^13^ observed that the lower extremity muscles must have sufficient strength to perform functional tasks, and patients with knee osteoarthritis are more likely to have thigh muscle weakness than normal adults. This indicates lower muscle strength and pain and knee A variety of clinical features including joint function are related. In screening patients of the same age and size, factors such as mobility, gender, BMI, and structural damage were objectively measured. It was found that the patient's pain level and the difficulty of knee movement were positively related to the strength of the quadriceps and hamstring muscles. Multiple studies have shown that increasing the strength of the quadriceps and hamstring muscles may help reduce pain and exercise in daily life, and improve mobility in patients with advanced knee osteoarthritis^14^, ^15^, ^16^.

This experiment also found that, excluding other influencing factors, the patients measured their peak torque (PT) through the isokinetic strength test system before the quadriceps and hamstring muscle strength surgery, and the results were significantly different from the healthy side. And it indicating that the knee joint extension and flexion muscle strength is significantly reduced. In addition, the isokinetic strength test showed that the TW of knee extension and knee flexion also decreased, showing that the work capacity and response ability of the muscles around the knee joint were significantly reduced. The knee joint HSS function scores are also lower than those on the healthy side. These research indicators have shown that the muscle strength around the knee joint is significantly lower than that on the healthy side, and the decline in joint function directly reduces the patient's daily activities and significantly affects quality of life.

### *Advantages of* *TKA* *for Treating* *KOA*

The treatment of KOA is mainly conservative and surgical treatment. Late‐stage patients often choose to undergo total knee replacement surgery after conservative ineffectiveness. TKA is a relatively thorough alternative for the purpose of relieving pain, correcting the knee lower limb force lines, and improving the motor function of the joints^17^. Ferket *et al*.^18^ conducted a randomized controlled study of five patients receiving an alternative to surgical and non‐surgical treatment. The trial population mainly included patients with severe symptoms, and the average quality of life was low. The results showed that the patient's pain and physical limitations were greatly improved, and a significant improvement in quality of life was found during long‐term follow‐up.

Shan *et al*.^19^ did a lot of related research on the quality of life of patients after knee replacement. These studies have shown that, whether in mid‐term or long‐term follow‐up, the study subjects were more relevant to general patients after total knee replacement surgery. The quality of life has improved significantly. Lai *et al*.^20^ found that elderly patients are usually willing to undergo surgery because their knees lose their knee space due to degenerative changes and deformities of the lower limbs, which severely limits their knee mobility and daily life activities, and the surgery often improves quality of life. Improving the quality of life has often become the primary pursuit of patients. TKA surgery is to replace the damaged limbs with artificial knee prostheses and pads, which can relieve long‐term joint pain symptoms, gradually increase the range of activities, and restore knee function and movement. In addition, there are reports in the literature that TKA is also used to treat some severe limb deformities that are biased towards the lower limb force line. These deformities are mainly secondary to fracture deformities and osteoarthritis caused by bone metabolic diseases^21^.

Hazratwala *et al*.^22^ conducted a large number of retrospective cohort studies on patients with TKA for joint deformities. These patients often have more serious deformities. The patient's knee stiffness for a long time causes the knee joint to lose its motor function, so it is necessary to restore the patient's lower limb force spool and balance soft tissue. The mechanical axis balances the soft tissues. They reported that the majority of patients undergoing TKA have a good recovery prognosis in the short to medium term, and the results of the surgery have reached consensus. This study also observed that patients who were given advanced knee replacement surgery had a follow‐up period of up to 6 months. As the knee deformity was corrected, the knee function also improved significantly compared to before surgery, and the HSS score of the affected knee increased significantly. At 6 months, the healthy side has been approached or even reached. The bilateral knee function is relatively balanced, indicating that TKA has a significant effect on the treatment of advanced KOA, and the quality of life of patients has improved accordingly.

### *Knee Muscle Strength Recovery after* *TKA*

At present, there is a large amount of literature showing that there is a significant correlation between changes in muscle strength around the knee joint and knee motor function after knee replacement^23^, ^24^, ^25^, ^26^. As mentioned earlier, this study also found that preoperative KOA patients' knee extension and flexion muscle strength will significantly decrease, which will affect the knee function. However, after TKA treatment, the isokinetic strength test of the knee joint around the surgical side showed that with time the isokinetic strength at 3 and 6 months after surgery was significantly better than before. And the muscle strength around the knee increased rapidly from 3 months to 6 months, and it approached the healthy side level 6 months after surgery. It indicates that the period of 3 months to 6 months after surgery is the best recovery time.

Castorina *et al*.^27^ thought that if the knee joint muscle strength recovery is not good after the knee joint operation, the knee joint function will also be affected, which will also affect the postoperative recovery. Piva *et al*.^28^ found that changes in knee extension and flexion after knee replacement have a significant relationship with resuming daily exercise. If knee extension and knee flexion are not well restored, it will lead to gait in daily walking and stair climbing. The rate of activity was significantly lower than in normal middle‐aged and elderly people. Iwata *et al*.^29^ evaluated gait function, bilateral quadriceps strength, and pain status in 49 patients who planned to undergo unilateral TKA. He observed from the time before surgery to 3 weeks after surgery that there was a clear correlation between the patient's gait function and the strength of the quadriceps. And it is necessary to reconsider the patient's rehabilitation plan by measuring the strength of the quadriceps muscle on the surgical side. Cavanellas *et al*.^30^ evaluated the strength of the quadriceps and hamstrings in 100 patients at a rate of 60°/s, and found that changes in the strength of quadriceps and hamstrings after TKA improved joint disease and it is important for a rehabilitation plan after surgery.

### *Application of Isokinetic Muscle Strength Test System*

The isokinetic strength test system used in this study can accurately assess the muscle strength of the knee joint. In our research, the applied angular velocity is 60°/s, because under the condition of 60°/s more muscle fibers are required to participate in the unit time during the test, which reflects the absolute strength of the muscles and is more in line with the working state of the muscles in daily life. High‐speed tests such as 180°/s angular velocity test focus more on observing muscle endurance. Among the isokinetic strength test indicators, the effect of knee extension on the clinical symptoms of KOA patients is greater than that of knee flexion, and the relative peak work of knee extension at 60°/s can best reflect the clinical symptoms of KOA patients. It can be used as an index to quantitatively evaluate the clinical symptoms of KOA patients.

Perrin *et al*.^31^ believe that the angular velocity of 60°/s is the safest angular velocity for the patellofemoral joint, and it is especially suitable for evaluating the peak knee torque of the elderly, and should be used preferentially. In addition, knee joint rehabilitation with an angular velocity of 60°/s can achieve the purpose of improving muscle strength and speeding up muscle strength recovery, enabling patients to resume functional activities as much as possible. Related scholars^32^, ^33^ have shown that isokinetic muscle strength test can not only accurately measure muscle strength, but can also regulate the control of muscle function by nerves and enhance joint stability, flexibility, and coordination of movements. It is one of the most valuable tools for analyzing and improving the muscle strength and the range of motion of the joint. It can evaluate and train the six major joints of the shoulder, elbow, wrist, hip, knee, and ankle.

In this study, the muscle strength around the knee was tested on the affected side and the healthy side at the same time. By comparing the test results and statistical analysis, it was found that the extensor knee muscle strength can reach the contralateral level after surgery, and the same is true for the knee flexion muscle strength. Relevant research reports have also confirmed that the muscle strength of the quadriceps and hamstring muscles will increase after knee replacement surgery. The recovery state is better at 6 months, and the changes of the extensor muscles are more than the flexion muscles significantly^34^. Therefore, the muscle strength improvement after TKA should be more focused on the extensor muscle training than the flexor knee muscle strength, so as to achieve the healthy extensor strength and maintain the bilateral knee joint muscle balance.

In addition to measuring the knee extension and flexion strength, the isokinetic strength test system can also directly test the knee extension and flexion TW. TW can directly display the knee joint reaction ability and the strength and force of the muscles around the knee joint. There is a greater relationship between the strength of the surrounding muscle group and the distance of the acting force. The total amount of knee extension and flexion work is one of the main indicators for judging the muscle function around the knee joint. It also indirectly reflects the size of the knee extension and flexion strength. The HSS scale has a good accuracy for the evaluation of the improvement of knee function before and after knee replacement surgery and the function of each period of replacement surgery. It has obvious advantages for evaluating the changes of knee joint function in the early stage after TKA operation, which can better evaluate the movement status of femoral tibial joint and patellofemoral joint, and improve the accuracy of the results of this subject.

Tolk *et al*.^35^ conducted a randomized controlled study using HSS scores for follow‐up of patients after replacement, and used self‐reported questionnaires on physical function, quality of life, and psychological factors when patients met expectations during follow‐up. Measurements were performed and it was found that the majority of patients obtained measurements when they achieved the desired goal after surgery.

This study found that applying HSS scores to this survey of patients was reliable, effective, and considered a high‐quality tool for achieving expectations. It was also found that patients had significant differences in total knee extension and flexion before and after TKA. Before and after operation, 1 month after operation, 3 months after operation, and 6 months after operation, the total work of the knee joint was measured and compared, and the total work of lateral extension and knee flexion was found to be less than that of the healthy side. By 6 months after surgery, the total knee extension effort of the surgical knee had approached the contralateral level, indicating that the knee joint reaction capacity had improved significantly. In the same way, follow‐up HSS score can be obtained that the HSS score has improved with time after surgery, and it has approached the healthy side level after 6 months. The patient's knee function of the operation side has no obvious obstacles and can complete daily activities, significantly improving the quality of life of patients. In this study, because the isokinetic muscle strength test system has high accuracy and good patient acceptability, it only needs to be performed when the patient returns to the hospital for review. According to the specific situation of the patient, the next rehabilitation program is given, which improves the motivation of the experimental subjects and compliance.

### *Importance of Functional Exercise after* *TKA* *to Restore Muscle Strength*

This study shows that the affected knee joint function can be improved after knee replacement, but patients also need functional exercises to increase the muscle strength around the knee joint after knee replacement, which is conducive to the faster recovery of knee function in patients. However, after replacement, patients often reduce their daily activities due to pain and other reasons. Most patients in the early stage were mainly active in bed and simple walking.

Daugaard *et al*.^36^ followed up patients with TKA for about 5 years and compared them with healthy people of the same age. They found that patients who have undergone TKA surgery prefer to sit still for a long time and tend to avoid prolonged activities. In addition, the number of short walks of patients after surgery was relatively reduced by two times compared with normal peers. This indicates that patients after TKA surgery generally do not affect health‐related activities. But it may affect some specific behaviors, possibly due to pain or functional limitations. This indicates that patients after TKA surgery generally do not affect health‐related activities. But it may affect some specific behaviors, possibly due to pain or functional limitations.Therefore, in order to make the postoperative knee joint activity recovery better, attention should be paid to instruct the patient to actively recover. Jakobsen *et al*.^37^ observed the quadriceps muscle activity of patients undergoing strength training after TKA, and concluded that the effect of functional training in patients at home is simpler than the strength training in earlier machines. Strength training can lead to higher peak quadriceps muscle activity. Therefore, in the early stage after the replacement, the patient can use a simpler form of strength training according to his own condition. Oktas *et al*.^38^ believe that although TKA surgery is the gold standard for the treatment of end‐stage KOA, surgery is only one aspect of improving knee function, and the pain and edema of the knee joint must be controlled immediately after surgery.

We should formulate a rehabilitation plan to enhance the muscle strength around the knee joint, which can increase the success rate of patients' surgery. Exercise‐specific therapy is also required after a regular rehabilitation plan, which can extend the life of the prosthesis, prevent joint prosthesis from loosening, and avoid undue mechanical stress. In addition to traditional rehabilitation methods to accelerate the functional recovery of patients after TKA, neuromuscular electrical stimulation and higher intensity strength training programs should also be considered. If optimal results are still not achieved, exercise in water and intensive training can be performed^39^.

Bade *et al*.^40^ concluded that after performing a high‐intensity rehabilitation plan for postoperative patients, compared with low‐intensity exercise therapy, high‐intensity exercise therapy after TKA can improve short‐ and long‐term function and muscle strength of knee extension, and experiments have shown that higher intensity exercise therapy does not cause any complications. Therefore, the key role of rehabilitation exercise after TKA for knee joint recovery is self‐evident, and even directly determines the success of TKA surgery, which should be paid attention. Whether it is general strength training or high‐intensity exercise therapy after surgery, it is especially important to develop a specific rehabilitation plan based on the patient's specific condition after the surgery.

The ISOMED2000 instrument can detect the specific conditions of the knee extensor and flexor muscle strength of the patient, and can use isokinetic training to increase muscle strength. Similarly, the peak torque of the extensor and flexor force is often used as the main test target for muscle strength changes, combined with other rehabilitation therapies to promote postoperative recovery.

### *Restore the Balance of Knee Flexion and Extensor Strength*

Stastny *et al*.^41^ tested athletes' knee flexion and extensor muscle strength to determine whether athletes are at risk of injury to muscles around the knee joint. The traditional concentric rope‐to‐concentric quadriceps ratio (H/Q) was used to describe the assessment of the ipsilateral imbalance of the knee flexors and extensors. The study found that the relationship between knee flexion and knee extension strength is an important indicator to assess the risk of lower limb injuries. Especially when there is an imbalance in hamstring strength, the risk of knee injury increases. Therefore, in order to increase the strength, elasticity and functional performance of the hamstring, it is necessary to test and train the hamstring at different eccentric speeds.

At higher speeds, testing of knee extensor and flexor strength may be more ecologically effective. Therefore, such a strength test scheme should include footwork that gradually increases speed. If isokinetic testing shows a large difference between the strength of the eccentric hamstrings and the concentric quadriceps, the difference should be reduced through training. For this reason, especially in sports environments, reducing the knee extensor strength must not compensate for weak knee flexors at the expense of a weaker H/Q ratio.

Gradually and concentratedly increasing the strength of the knee flexor muscles can make the rope muscles stronger at higher speeds, especially compared to the quadriceps. Therefore, if isokinetic testing shows a certain degree of hamstring weakness, training interventions may be necessary to increase the strength of the hamstring muscles. Therefore, to maintain the function and stability of the knee joint, rehabilitation should be performed through reasonable functional exercises to achieve the desired effect.

### *Outlook*

In addition to formulating a rehabilitation plan for the affected side of the patient after knee replacement, the secondary knee of the healthy side should also pay attention to secondary prevention or limit the further progress of early knee osteoarthritis. As a high‐risk group, pain and functional decline on the surgical side of the patient have a great impact on the patient in many ways. Secondary prevention of the healthy side limb is also key to effectively preventing the further development of the disease. Appropriate guidance should be provided according to the patient's personal risk factors, which can effectively limit the development of early osteoarthritis, improve the quality of life of patients, and reduce the incidence of disability, while avoiding surgical pain and reducing the economic pressure on patients.

In summary, we found that after TKA, the knee muscle strength and total knee energy of the patients improved significantly 3 and 6 months after the operation. Following this, the HSS score of knee joint function changed significantly, reaching or approaching the healthy side level 6 months postoperatively. Therefore, TKA has a good effect on the treatment of end‐stage KOA patients and has a significant impact. However, surgery alone cannot help patients fully recover their knee joint function. The isokinetic muscle strength test system can be used to monitor the muscle strength around the knee joint, especially 3–6 months after surgery. A good rehabilitation plan should be formulated according to the specific situation of the patient, and knee rehabilitation training should be strengthened to make the knee function on the surgical side faster and more scientific, and help the patient improve the quality of life.

## Conclusion

Knee joint muscle strength and knee joint function after TKA are significantly improved compared with their function before operation, which is of great significance for the treatment of knee osteoarthritis. The isokinetic muscle strength test system has the advantages of safety, accuracy, repeatability, and easy operation. It is a good method to evaluate the muscle strength and function of the knee around the time of knee replacement.
